# Association with severe and treatment-resistant depression among patients with inflammatory joint disease. Nationwide nested case-control study in Swedish registers

**DOI:** 10.1192/j.eurpsy.2022.313

**Published:** 2022-09-01

**Authors:** P. Brenner, J. Askling, P. Stang, L. Brandt, D. Hägg, J. Reutfors

**Affiliations:** 1 Karolinska Institutet, Medicine Solna, Stockholm, Sweden; 2 Janssen, Global Services, Titusville, United States of America

**Keywords:** Rheumatology, Inflammatory joint disease, Epidemiology, Depression

## Abstract

**Introduction:**

Treatment resistant depression (TRD) and severe depression (SD) are common among patients with depression. Patients with inflammatory joint disease (IJD) are at higher risk for developing depression compared to the general population; however, the risk for SD or TRD is not known.

**Objectives:**

To examine the odds of patients with IJD for developing SD and TRD compared to non-severe and non-TRD depression.

**Methods:**

This case-control study was nested within a cohort of patients with incident depression (n=443,384) identified in nationwide Swedish registers 2006-2018. Patients with SD (n=42,975) were identified through the ICD-10 code specifier, through psychiatric hospitalization and/or through suicide attempts. Patients who started a third consecutive treatment for depression were identified with TRD (n=33,830). Each patient was matched with five non-SD - or non-TRD - patients by sociodemographics and year of cohort entry. Crude and adjusted odds ratios (aOR) were calculated by conditional logistic regression with regard to a history of any IJD and specific IJDs prior to depression onset.

**Results:**

Among patients with depression, those with a history of IJD did not have higher odds for developing SD (aOR 1.09 (95%CI 1.00-1.20)) or TRD (aOR 1.03 (0.93 - 1.14)) compared to patients without IJD. A history of rheumatoid arthritis was associated with a significantly higher odds for SD among patients aged 18-29 (aOR 1.55 (1.01-2.36)) and for TRD among patients aged 30-49 (aOR 1.33 (1.05-1.67).

**Conclusions:**

Overall, no association was observed between history of IJD and developing SD/TRD; with the exception of younger age strata in rheumatoid arthritis.

**Disclosure:**

PB, JA, DH, LB, and JR are affiliated to or employees at The Unit for Clinical Epidemiology, Karolinska Institutet, which receives grants from several entities (pharmaceutical companies, regulatory authorities, contract research organizations) for the per

